# Prognostic value of serum lactate level for mortality in patients with acute kidney injury

**DOI:** 10.1186/s40001-024-01886-5

**Published:** 2024-05-22

**Authors:** Meng Chen, Dezhao Lv

**Affiliations:** Department of Rehabilitation, The Second Affiliated Hospital and Yuying Children’s Hospital of Wenzhou Medical University, Wenzhou, 325000 Zhejiang China

**Keywords:** Lactate, Acute kidney injury, Mortality, AKI

## Abstract

**Background:**

Serum lactate is associated with mortality in diverse kinds of patients. This study aimed to investigate whether serum lactate level may independently predict mortality in acute kidney injury (AKI) patients.

**Methods:**

A total of 4461 AKI patients were collected from the Medical Information Mart for Intensive Care (MIMIC III) database and followed up for 365 days. According to serum lactate tertiles, participants were divided into three groups (Q1–Q3) by: Q1 ≤ 1.60 mg/dl, Q2 = 1.61–2.70 mg/dl, and Q3 ≥ 2.71 mg/dl. We calculated the hazard ratio (HR) and 95% confidence intervals (Cls) for mortality across each tertile of lactate by using the Q1 as reference and constructed four models to adjust for the HR of mortality.

**Results:**

Nonsurvivors had significantly higher lactate compared with patients in the survival group. Mortality rate gradually elevated with the increase in serum lactate level (Q1: 29.30%, Q2: 33.40%, Q3: 37.40%). When compared with Q1 after adjustment of all confounders, the HRs of Q3 still was 1.20 (95% Cl 1.05–1.37).

**Conclusions:**

This study demonstrated that high serum lactate levels were an independent predictor of mortality in AKI patients.

## Introduction

Acute kidney injury (AKI) is a serious and common syndrome. AKI is often associated with increased mortality and poor prognosis in patients, especially in critical illness [1, 2]. In addition, survivors often fail to completely recover renal function and need renal replacement therapy (RRT) [3], which brings a large social and economic burden [4]. Considering the high incidence of AKI, a growing number of observational studies have been devoted to discovering the clinical biomarkers to predict mortality in AKI patients. However, the clinical application of these biomarkers to predict AKI outcome remains unsatisfactory. Thus, it is essential to discover a new biomarker to help develop novel treatments and diminish the risk of death.

Previous studies have suggested that serum lactate level had a relationship with mortality in populations of patients including those with sepsis, cancer, focal ischemic conditions, acute cardiorespiratory insufficiency and low-flow states [5–9]. It has been reported that high serum lactate was generally associated with tissue hypoxia [10]. Meanwhile, an increased lactated level is also observed in the setting of decreased lactate clearance owing to kidney disease [11]. We hypothesize that serum lactate may be a marker to predict the mortality in AKI patients. Nevertheless, it is surprising that few studies have investigated the relationship between mortality and lactate in AKI patients. In our study, we examined whether serum lactate levels may be capable of predicting mortality in patients with AKI.

## Methods

### Data source

This study was based on the publicly and freely accessible database named Medical Information Mart for Intensive Care III (MIMIC-III, version 1.4). This database comprises information associated with over 40,000 distinct hospital admissions at the Beth Israel Deaconess Medical Center (Boston, USA) form 2001 to 2012 [12]. The establishment of this database was approved by the Institutional Review Boards of the Beth Israel Deaconess Medical Center and Massachusetts Institute of Technology. To apply for permission to access the database, we passed the National Institutes of Health’s web-based training course named ‘Protecting Human Research Participants’ (Certification number: 41624341).

**Fig. 1:**
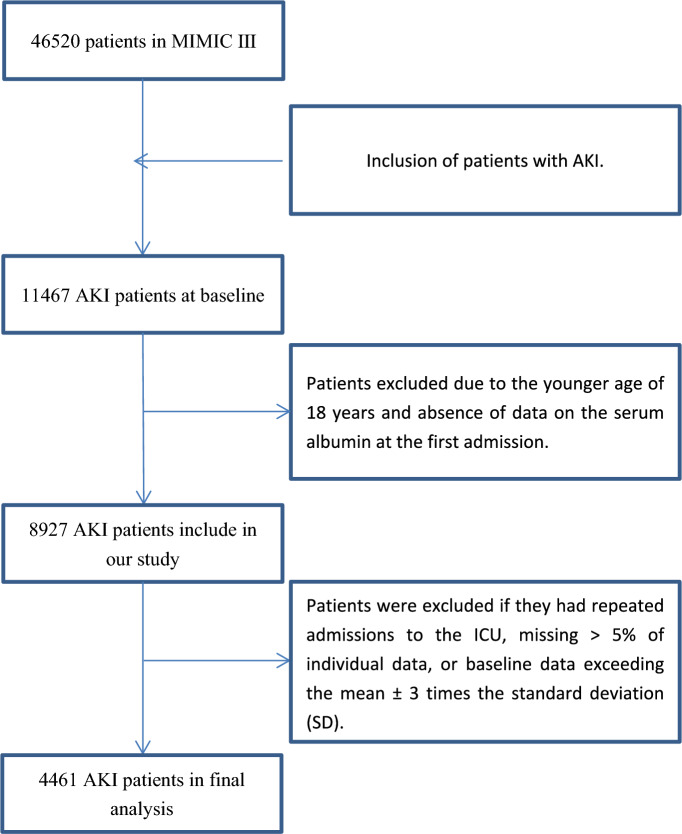
The flowchart of inclusion and exclusion procedure

A total of 11,467 distinct patients with AKI were recorded in the database. In this study, we collected 4461 patients who were older than 18 years and the primary diagnosis was AKI at ICU admission. AKI was defined according to ICD-9 diagnostic code containing the terms “acute renal failure”. The individuals were excluded if (1) absence of data on the serum lactate level at the first admission; (2) > 5% of the required data were missed; (3) baseline data values exceeded the mean ± 3 times the standard deviation (SD) (Fig. 1).

### Data extraction

Data on the clinical parameters, laboratory parameters, clinical scores and comorbidities were extracted from MIMIC-III using the Structured Query Language (SQL) with PostgreSQL tools (version 12.0). The clinical parameters included age, sex, heart rate, respiratory rate, temperature, systolic blood pressure (SBP), diastolic blood pressure (DBP), mean arterial pressure (MAP), percutaneous oxygen saturation (SPO_2_), vasoactive drug, and RRT. The laboratory parameters included glucose, white blood cell, platelet, sodium, potassium, blood urea nitrogen (BUN), bicarbonate, chloride, anion gap, and creatinine. Comorbidities including congestive heart failure, hypertension, diabetes, stroke, chronic renal disease, chronic liver disease and malignancy were also extracted. Furthermore, the Simplified Acute Physiology Score II (SAPS II), Sequential Organ Failure Assessment (SOFA) and Glasgow Coma Scale (GCS) were calculated for each individual. The patient’s data at first admission were used as the baseline data. The primary outcome was 365-day mortality and secondary outcomes were 30-day mortality, 90-day mortality and 270-day mortality.

### Statistical analysis

Descriptive statistics such as mean ± SD and percentage were calculated for continuous and categorical variables, respectively. To obtain a deeper understanding of the association between serum lactate and mortality in AKI, all patients were divided into three groups by serum lactate tertiles statistically. In this study, tertiles were categorized separately as follows: Q1 ≤ 1.60 mg/dl, Q2 = 1.61–2.70 mg/dl, and Q3 ≥ 2.71 mg/dl. Continuous variables were presented as the mean ± SD and compared using one-way ANOVA or Mann–Whitney *U* test as appropriate. Categorical variables were summarized as percentages and compared using *X*^2^ test. The relationship between lactate and mortality was explored using Cox regression for univariate and multivariate analyses. We were based on Kaplan–Meier analysis and log-rank test to construct survival curves. The results were expressed as hazard ratios (HRs) with 95% confidence intervals (Cls). All statistical analyses were performed using SPSS 21.0 (SPSS Inc, Chicago, IL). Values of *P* < 0.05 were defined as statistically significant.

## Results

### Baseline characteristics of AKI patients

A total of 4461 eligible participants with AKI were enrolled in this study. The mean age of these patients at admission was 65 years, of which 2626 (58.9%) were male. The characteristics of survivors and nonsurvivors are presented in Table 1. There was no significant difference in gender, sodium, chloride, and creatinine between survivors and nonsurvivors. Compared with survivors, patients in the nonsurvival group were older and had significantly higher heart rate, lactate, white blood cell, potassium, BUN, anion gap, SOFA, and SAPS II score, whereas temperature, blood pressure, SPO_2_, glucose, platelet, sodium, bicarbonate, chloride, and GCS score were significantly lower. In addition, the nonsurvivors tended to report a history of congestive heart failure.

**Table 1:** Baseline characteristics of the study population

Characteristics	Survivors	Nonsurvivors	P value
Clinical parameters			
Age (years)	62.43 ± 15.74	69.48 ± 13.81	< 0.001
Sex (male) [*n* (%)]	1423 (58.10)	1203 (59.80)	0.142
Heart rate (mean ± SD)	88.75 ± 16.89	90.18 ± 17.82	0.006
Respiratory rate (mean ± SD)	19.90 ± 4.37	20.80 ± 4.68	< 0.001
Temperature (mean ± SD) (℃)	36.91 ± 0.70	36.67 ± 0.81	< 0.001
SBP (mean ± SD)(mmHg)	117.73 ± 16.73	111.69 ± 16.53	< 0.001
DBP (mean ± SD)(mmHg)	61.01 ± 11.04	57.02 ± 10.27	< 0.001
MAP (mean ± SD)(mmHg)	77.22 ± 11.39	73.40 ± 10.72	< 0.001
SPO_2_ (%)	97.07 ± 2.23	96.52 ± 3.63	< 0.001
Vasoactive drug [*n* (%)]	1060 (43.30)	1271 (63.10)	< 0.001
RRT used [*n* (%)]	130 (5.30)	153 (7.6)	0.001
Laboratory parameters (mean ± SD)			
Lactate (mg/dl)	2.25 ± 1.33	2.78 ± 1.70	< 0.001
Glucose (md/dl)	161.38 ± 81.75	153.00 ± 66.14	< 0.001
White blood cell (10^9^/l)	12.81 ± 6.88	14.59 ± 15.19	< 0.001
Platelet (10^9^/l)	221.54 ± 122.17	210.56 ± 136.28	0.005
Sodium (mmol/l)	138.26 ± 5.43	137.99 ± 6.15	0.119
Potassium (mmol/l)	4.34 ± 0.72	4.46 ± 0.75	< 0.001
BUN (md/dl)	40.17 ± 30.00	46.83 ± 31.30	< 0.001
Bicarbonate (mmol/l)	21.70 ± 4.89	21.15 ± 5.16	< 0.001
Chloride (mmol/l)	105.16 ± 6.77	104.85 ± 7.26	0.150
Anion gap (mmol/l)	15.89 ± 4.13	16.73 ± 4.01	< 0.001
Creatinine (mg/dl)	2.23 ± 1.91	2.30 ± 1.45	0.234
Clinical scores (mean ± SD)			
SOFA	6.08 ± 3.16	7.89 ± 3.86	< 0.001
GCS	13.57 ± 2.84	13.17 ± 3.17	< 0.001
SAPS II	40.51 ± 13.18	52.17 ± 14.90	< 0.001
Comorbidity [*n* (%)]			
Congestive heart failure	555 (22.70)	667 (33.10)	< 0.001
Hypertension	504 (20.60)	429 (21.30)	0.290
Diabetes	868 (35.50)	676 (33.6)	0.100
Stroke	76 (3.10)	56 (2.80)	0.294
Chronic renal disease	580 (23.70)	543 (27.00)	0.007
Chronic liver disease	257 (10.50)	295 (14.70)	< 0.001
Malignancy	144(29.40)	345(70.60)	< 0.001

*BUN* blood urea nitrogen, *DBP* diastolic blood pressure, *MAP* mean arterial pressure, *RRT* renal replacement therapy, *SPO2* percutaneous oxygen saturation, *SAPS II* Simplified Acute Physiology Score II, *SBP* systolic blood pressure, *SOFA* Sequential Organ Failure Assessment, *GCS* Glasgow Coma Scale

### Characteristics of subject stratified by serum lactate

Table 2 shows baseline characteristics of patients divided into the three lactate levels, where Q1 was up to 1.60 mg/dl, Q2 was 1.61–2.70 mg/dl, and Q3 was of at least 2.71 mg/dl. Patients with higher lactate tended to have a history of vasoactive drug used, RRT, and chronic liver disease. Meanwhile, these patients also had higher heart rate, serum glucose, white blood cell, potassium, anion gap, and severity scores (i.e., SOFA and SAPS II). There was no significant difference in gender, DBP and chloride between different groups.

**Table 2:** Baseline characteristics of patients according to the tertiles of lactate

Characteristics	Lactate level			*P* value
	Q1 ≤ 1.60 mg/dl	Q2 = 1.61–2.70 mg/dl	Q3 ≥ 2.71 mg/dl	
Number	1570	1489	1402	
Clinical parameters				
Age (years)	65.32 ± 14.88	66.50 ± 15.02	65.00 ± 16.03	0.019
Sex (male) [*n* (%)]	891 (56.80)	893 (60.00)	842 (60.10)	0.107
Heart rate (mean ± SD)	86.41 ± 16.23	88.78 ± 16.55	93.39 ± 18.54	< 0.001
Respiratory rate (mean ± SD)	19.96 ± 4.41	20.09 ± 4.45	20.93 ± 4.69	< 0.001
Temperature (mean ± SD) (℃)	36.85 ± 0.69	36.79 ± 0.71	36.77 ± 0.88	0.014
SBP (mean ± SD)(mmHg)	118.44 ± 17.76	113.86 ± 15.75	112.38 ± 16.48	< 0.001
DBP (mean ± SD)(mmHg)	59.53 ± 11.13	58.90 ± 10.69	59.19 ± 10.79	0.277
MAP (mean ± SD)(mmHg)	76.32 ± 11.70	74.73 ± 10.57	75.40 ± 11.39	< 0.001
SPO_2_ (%)	96.86 ± 2.26	97.05 ± 2.38	96.54 ± 4.00	< 0.001
Vasoactive drug [*n* (%)]	662 (42.20)	763 (51.20)	906 (64.60)	< 0.001
RRT used [*n* (%)]	100 (6.40)	70 (4.70)	113 (8.10)	0.001
Laboratory parameters (mean ± SD)				
Glucose (md/dl)	142.03 ± 56.68	157.42 ± 78.25	175.24 ± 85.61	< 0.001
White blood cell (10^9^/l)	12.48 ± 12.18	13.89 ± 8.49	14.58 ± 13.08	< 0.001
Platelet (10^9^/l)	236.45 ± 127.93	218.83 ± 135.30	192.04 ± 118.40	< 0.001
Sodium (mmol/l)	138.12 ± 5.22	137.91 ± 6.00	138.40 ± 6.09	0.075
Potassium (mmol/l)	4.33 ± 0.72	4.39 ± 0.73	4.46 ± 0.74	< 0.001
BUN (md/dl)	44.85 ± 32.04	44.07 ± 31.84	40.36 ± 27.83	< 0.001
Bicarbonate (mmol/l)	22.55 ± 5.36	21.64 ± 4.79	20.03 ± 4.51	< 0.001
Chloride (mmol/l)	104.90 ± 6.65	104.96 ± 7.23	105.22 ± 7.12	0.406
Anion gap (mmol/l)	15.18 ± 3.72	15.83 ± 3.86	17.95 ± 4.21	< 0.001
Creatinine (mg/dl)	2.38 ± 2.09	2.17 ± 1.54	2.21 ± 1.40	0.001
Clinical scores (mean ± SD)				
SOFA	5.78 ± 2.95	6.59 ± 3.36	8.48 ± 3.96	< 0.001
GCS	13.59 ± 2.69	13.40 ± 2.95	13.16 ± 3.35	0.001
SAPS II	41.61 ± 13.43	45.03 ± 14.45	51.21 ± 16.00	< 0.001
Death, *n* (%)				
30-day	306 (24.60)	388 (31.20)	549 (44.20)	< 0.001
90-day	437 (26.90)	533 (32.80)	653 (40.20)	< 0.001
270-day	562 (29.00)	635 (32.80)	738 (38.10)	< 0.001
365-day	589 (29.30)	672 (33.40)	752 (37.40)	< 0.001
Comorbidity [*n* (%)]				
Congestive heart failure	511 (32.50)	394 (26.50)	317 (22.60)	< 0.001
Hypertension	418 (26.60)	311 (20.90)	204 (14.60)	< 0.001
Diabetes	580 (36.90)	518 (34.80)	446 (31.80)	0.013
Stroke	50 (3.20)	36 (2.40)	46 (3.30)	0.316
Chronic renal disease	483 (30.80)	382 (25.70)	258 (18.40)	< 0.001
Chronic liver disease	130 (8.30)	199 (13.40)	223 (15.90)	< 0.001
Malignancy	150 (9.60)	173 (11.60)	166 (11.80)	0.084

*BUN* blood urea nitrogen, *DBP* diastolic blood pressure, *MAP* mean arterial pressure, *RRT* renal replacement therapy, *SPO2* percutaneous oxygen saturation, *SAPS II* Simplified Acute Physiology Score II, *SBP* systolic blood pressure, *SOFA* Sequential Organ Failure Assessment, *GCS* Glasgow Coma Scale

### Association between lactate and mortality

**Fig. 2:**
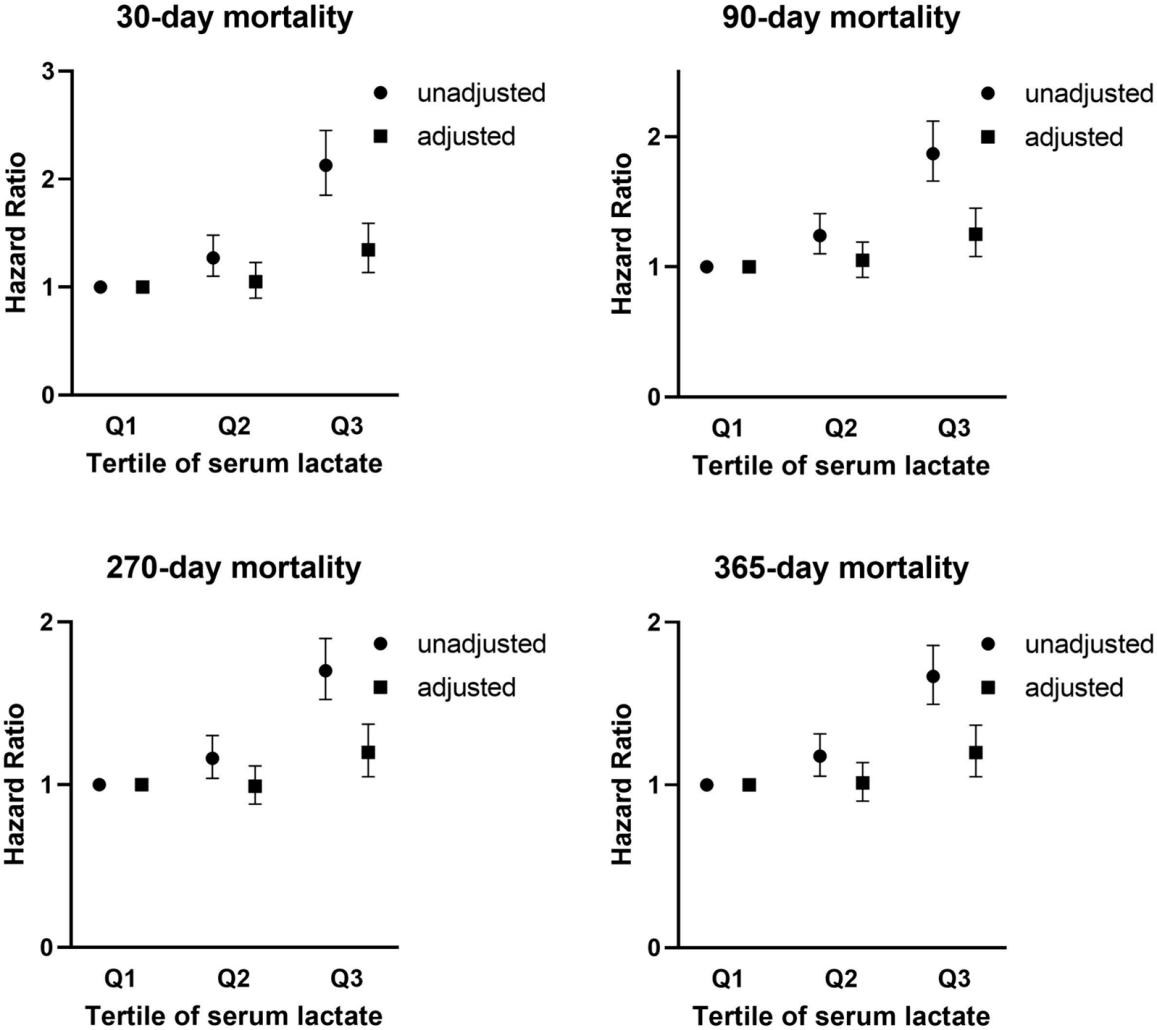
Unadjusted and adjusted hazard ratio for lactate at different periods. Unadjusted is univariate analysis. Adjusted is adjusted for age, sex, temperature, heart rate, respiratory rate, vasopressin used, SBP, DBP, SPO_2_, MAP, RRT, stroke, hypertension, congestive heart failure, diabetes, chronic renal failure, chronic liver disease, malignancy, glucose, white blood cell, platelet, sodium, potassium, bicarbonate, chloride, anion gap, creatinine, BUN, SOFA, GCS, SAPS II. *BUN* blood urea nitrogen, *DBP* diastolic blood pressure, *MAP* mean arterial pressure, RRT renal replacement therapy, *SPO*_*2*_ percutaneous oxygen saturation, *SAPS II* Simplified Acute Physiology Score II, *SBP* systolic blood pressure, *SOFA* Sequential Organ Failure Assessment, *GCS* Glasgow Coma Scale

**Fig. 3:**
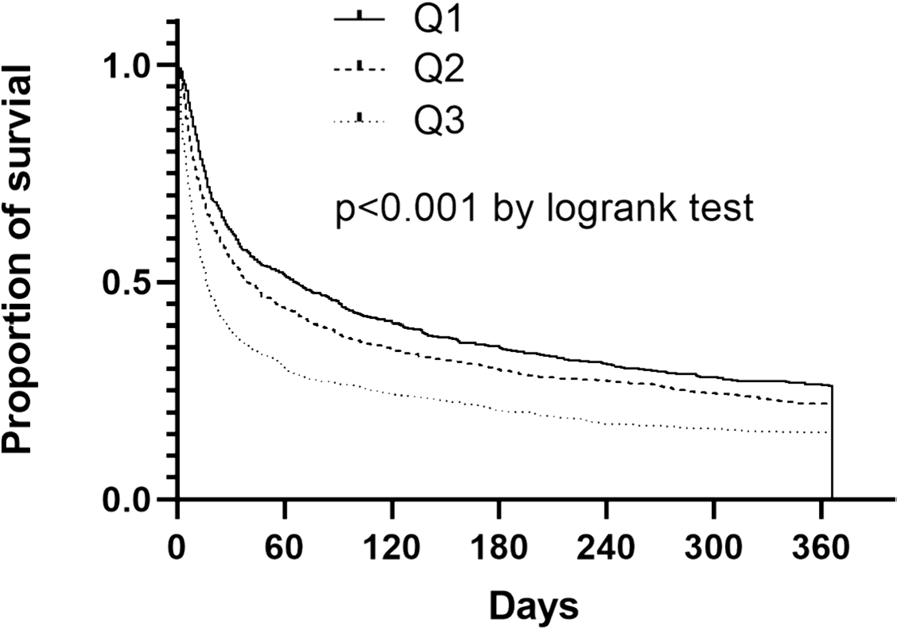
Kaplan–Meier curves reflecting survival probability according to tertiles of serum lactate. Patients with higher lactate level had a decreased proportion of survival

During the follow-up period, there were 549 deaths within 30 days, 653 within 90 days, 738 within 270 days, and 752 within 365 days. To examine whether increased serum lactate may be associated with the mortality rate of AKI patients, the Cox regression was used for mortality. Table 3 presents the HR of mortality in the four proportional hazard models. In unadjusted analysis, the HR for 365-day mortality was 1.18 (95% CI 1.05–1.32), and 1.67 (95% CI 1.50–1.86) for Q2 and Q3, respectively. Compared to Q1, the HR in Q3 still was 1.20 (95% CI 1.05–1.37, *P* = 0.007), indicating a significant association between serum lactate levels and 365-day mortality in the fully adjusted analysis (model 4). Furthermore, we examined the relationship between the serum lactate and 30-day, 90-day and 270-day mortality correspondingly by comparing the HR in the unadjusted and adjusted analysis in Fig. 2. The results indicated that patients with higher lactate levels had an increased risk of mortality. Figure 3 demonstrates the cumulative proportion of survival in groups of lactate by used the Kaplan–Meier analysis. These results indicated the HR of mortality gradually increased as the serum lactate level elevated.

**Table 3:** Hazard ratio (95% confidence interval) for 365-day mortality,

Tertiles of lactate	Unadjusted	*P* value	Model 1	*P* value	Model 2	*P* value	Model 3	*P* value	Model 4	*P* value
Q1	1	–	1	–	1	–	1	–	1	–
Q2	1.18 (1.05–1.32)	0.004	1.08 (0.97–1.21)	0.180	1.15 (1.03–1.28)	0.017	1.08 (0.96–1.20)	0.187	1.01 (0.90–1.13)	0.835
Q3	1.67 (1.50–1.86)	< 0.001	1.43 (1.27–1.60)	< 0.001	1.63 (1.46–1.82)	< 0.001	1.26 (1.11–1.43)	< 0.001	1.20 (1.05–1.37)	0.007

Model 1 is adjusted for clinical parameters (age, sex, temperature, heart rate, respiratory rate, vasopressin used, SBP, DBP, SPO_2_, MAP, RRT). Model 2 is adjusted for comorbidities (stroke, hypertension, congestive heart failure, diabetes, chronic renal failure, chronic liver disease, malignancy). Model 3 is adjusted for severity of illness (glucose, white blood cell, platelet, sodium, potassium, bicarbonate, chloride, anion gap, creatinine, BUN, SOFA, GCS, SAPS II). Model 4 included model 1, 2, and 3 covariates

*BUN* blood urea nitrogen, *DBP* diastolic blood pressure, *MAP* mean arterial pressure, *RRT* renal replacement therapy, *SPO*_*2*_ percutaneous oxygen saturation, *SAPS II* Simplified Acute Physiology Score II, *SBP* systolic blood pressure, *SOFA* Sequential Organ Failure Assessment, *GCS* Glasgow Coma Scale

## Discussion

In our study, we explored the association between the serum lactate and mortality in AKI patients. We found that nonsurvivors had significantly higher serum lactate than survivors. In addition, we found that lactate was positively related to mortality. Meanwhile, our results indicated that serum lactate level was an independent predictor of mortality in AKI patients. Therefore, lactate-guided therapy in AKI patients may be helpful to reduce mortality.

Our study showed that serum lactate was higher in the nonsurvival group compared with survivors, which was in line with previous studies. For example, one study conducted Sun et al. [13] by suggested that increased serum lactate was relationship with a higher mortality rate in cirrhosis with AKI. Another study showed that higher lactate was associated with higher mortality in critically ill patients in the emergency department [14]. Additionally, a retrospective study conducted by Liu et al. [5] indicated that patients with increased lactate had higher mortality than those with lower lactate and in the meanwhile serum lactate could accurately predicted mortality for patients with sepsis. Finally, a study conducted by Maher et al. [6] revealed that cancer patients with increased lactate had a higher risk of mortality. Taken together, these findings indicate that elevated serum lactate will probably increase mortality rate of patients.

The mechanisms underneath the relationship between serum lactate and mortality of AKI patients remain unclear. It has been reported that about 1500 mmol of lactate is daily produced primarily by highly glycolytic tissues in the human body, and is mainly metabolized in the liver and kidneys, which are rich in lactate dehydrogenase [15]. In physiological conditions, the generation and consumption of lactate are well-balanced [11]. Several researches have revealed that the increased lactate production was associated with microcirculatory oxygen delivery imbalance and capillary perfusion dysfunction [16, 17]. It is well known that the kidney exhibits lactate dehydrogenase dysfunction and hemodynamic changes in AKI. Thus, AKI may contribute to hyperlactatemia by affecting the production and consumption of lactate [13]. Therefore, elevated lactate can be viewed as a signal of tissue hypoxemia. A research conducted has reported that lactate could be observed as an indicator of oxygen debt. Meanwhile, the oxygen debt is closely associated with severity shock in mortality [18]. Another possible explanation of underlying mechanism may be associated to the involvement of acidosis. Our findings of increased lactate and anion gap levels indicated that metabolic acidosis may occur in AKI patients. Several studies showed that acidosis was positively associated with mortality in critically ill patients. For example, a study conducted by Gao et al. [19] reported that cirrhosis patients of metabolic acidosis had a high mortality rate and lactic acidosis was the worst prognosis of all types of metabolic acidosis. Another observational study showed that metabolic acidosis was closely associated with adverse kidney outcomes and mortality in patients with non-dialysis dependent chronic kidney disease [20]. Meanwhile, inflammation has been shown to play an important role in development of AKI [21]. Several studies have indicated that serum lactate was positively associated with inflammation [22, 23]. Therefore, serum lactate may affect the development of AKI by stimulating inflammation. Taken together, our study suggested that serum lactate level may play a vital role in the development of mortality in AKI patients, indicating that the higher lactate level was, the increased mortality occurred.

There are some limitations in our study. First, the information about patients stemmed from a single center. Therefore, subject selection bias might occur, limiting the generalization of our finding. Second, serum lactate level was only measured in patients admitted to the ICU once, which may have a biased influence on the results. Third, the measurement of serum lactate and anion gap does not completely reflect the extent of metabolic acidosis, and other indicators would be simultaneously measured for more comprehensive analysis. Fourth, because of lacking related data, we did not examine the modification of sepsis and shock, which might predict mortality in AKI patients.

## Conclusion

Our study indicated that lactate appeared to useful as a predictor of mortality in AKI patients and higher lactate was positively related to increased risk of mortality. However, our results need to be confirmed by large prospective studies.

## Data Availability

The datasets are available from the corresponding author on reasonable request.
